# Visualization of clonal expansion after massive depletion of cells carrying the bovine leukemia virus (BLV) integration sites during the course of disease progression in a BLV naturally-infected cow: a case report

**DOI:** 10.1186/s12977-022-00609-0

**Published:** 2022-11-03

**Authors:** Susumu Saito, Kazuyoshi Hosomichi, Meripet Polat Yamanaka, Tetsuya Mizutani, Shin-nosuke Takeshima, Yoko Aida

**Affiliations:** 1 Laboratory of Global Infectious Diseases Control Science, Graduate School of Agricultural and Life Sciences, The University of Tokyo, 1-1-1 Yayoi, Bunkyo-ku, Tokyo, 113-8657 Japan; 2 Viral Infectious Diseases Unit, RIKEN, 2-1 Hirosawa, Wako, Saitama 351-0198 Japan; 3 Institute of International Nutrition and Health, Jumonji University, 2-1-28 Sugasawa, Niiza, Saitama 352-8510 Japan; 4 Center for Infectious Diseases Epidemiology and Prevention Research, Tokyo University of Agriculture and Technology, 3-5-8 Saiwai-cho, Fuchu, Tokyo 183-8509 Japan; 5 Laboratory of Computational Genomics, School of Life Science, Tokyo University of Pharmacy and Life Sciences, 1432-1 Horinouchi, Hachioji, Tokyo 192-0392 Japan; 6 Department of Food and Nutrition, Faculty of Human Life, Jumonji University, 2-1-28 Sugasawa, Niiza, Saitama 352-8510 Japan

**Keywords:** Bovine leukemia virus, Integration, Provirus, Target enrichment high throughput sequencing system, Massive depletion, Clonal expansion, Lymphoma onset

## Abstract

Bovine leukemia virus (BLV) infects cattle, integrates into host DNA as a provirus, and induces malignant B-cell lymphoma. Previous studies have addressed the impact of proviral integration of BLV on BLV-induced leukemogenesis. However, no studies have monitored sequential changes in integration sites in which naturally infected BLV individuals progress from the premalignant stage to the terminal disease. Here, we collected blood samples from a single, naturally infected Holstein cow at three disease progression stages (Stage I: polyclonal stage, Stage II: polyclonal toward oligoclonal stage, Stage III: oligoclonal stage) and successfully visualized the kinetics of clonal expansion of cells carrying BLV integration sites using our BLV proviral DNA-capture sequencing method. Although 24 integration sites were detected in Stages I and II, 92% of these sites experienced massive depletion in Stage III. Of these sites, 46%, 37%, and 17% were located within introns of Refseq genes, intergenic regions, and repetitive sequences, respectively. At Stage III cattle with lymphoma, only two integration sites were generated de novo in the intergenic region of Chr1, and the intron of the *CHEK2* gene on Chr17 was significantly increased. Our results are the first to demonstrate clonal expansion after the massive depletion of cells carrying BLV integration sites in a naturally infected cow.

**Supplementary Information:**

The online version contains supplementary material available at 10.1186/s12977-022-00609-0.

## Introduction

The bovine leukemia virus (BLV) belongs to the family *Retroviridae* (genus *Deltaretrovirus*) and is closely related to human T-cell leukemia viruses (HTLV-1 and II) [1]. BLV infects cattle worldwide and induces enzootic bovine leukosis (EBL), the most common neoplastic disease in cattle [2–4]. Similar to other retroviruses, BLV integrates into the host genome as a provirus and induces lifelong infection. Approximately 70% of BLV-infected cattle are asymptomatic carriers; the remaining 30% of infected cattle show persistent lymphocytosis (PL), which is characterized by polyclonal expression of the non-neoplastic CD5^+^ B lymphocyte population. Less than 5% of cattle develop B-cell leukemia/lymphoma after a prolonged latency period [1].

Since virions such as BLV, HTLV-1, and HTLV-2 are particularly unstable, viruses are primarily transmitted by the transfer of a cell carrying an integrated provirus. Two steps characterize the course of these viral infections in vivo: a brief period of primary infection followed by chronic and persistent infection. Soon after the primary infection, viruses replicate via the infectious cycle or clonal expansion [5, 6]. In the case of the infectious cycle, viruses reach target lymphocytes by cell-to-cell transfer and establish by entry of viral single-stranded RNA, reverse transcription of the viral RNA, integration as a provirus into the host genome, expression of viral proteins, and budding of new virions. Thus far, two types of cell-to-cell contacts have been described as crucial for transmission: tight cell-cell contacts and cellular conduits [5, 7–9]. In the case of clonal expansion, mitotic division of cells harbors an integrated provirus [5].

The model experimentally infected by BLV showed that early infection is characterized by a massive depletion of proviral clones and polyclonal propagation. Since an antiviral immune response is quickly initiated, the vast majority of infected clones are depleted soon after infection [10]. Nevertheless, among the surviving proviruses, clone abundance is positively correlated with the proximity of the provirus to the transcribed region [10]. Between clonal expansion and cell proliferation, the survival of infected cells requires the silencing of viral expression before the immune response.

Human immunodeficiency virus type-1 (HIV-1) and HTLV-1 are preferentially integrated into actively transcribed genes, and cells infected by these viruses can undergo clonal expansion, which is enhanced by the integration of the provirus in actively transcribed areas of the genome [11, 12]. Like human retroviruses, BLV preferentially integrates into transcriptionally active genomic regions near transcriptional start sites and transcription factor-binding sites, and BLV integration accelerates the proliferation of infected cells [10, 13]. Indeed, the BLV provirus in BLV-induced B-cell lymphoma line BLSC-KU1 is integrated at a single site in the intron of the regulatory-associated protein of mTOR (*RPTOR*) gene, whereas in other B-cell lymphoma line BLSC-KU17, it is integrated at a single site in the intergenic region between *RTN4IP1* and *ATG5* [14]. Interestingly, previous research revealed that BLV provirus is preferentially integrated in the vicinity of cancer drivers that are affected via either premature transcription interruption or antisense dependent *cis*-perturbation. The same pattern already exists at early symptomatic stages of infection, indicating that provirus-dependent host gene perturbation contributes to the initial selection of the multiple clones characterizing the asymptomatic stage. Additional alterations are required in the clone to evolve into full-blown leukemia/lymphoma [13]. Likewise, other research groups have demonstrated that integration sites of two out of four EBL tumors are located next to genomic repeats, while some integration sites of the other two are located near transcribed regions, and the genetic profile of the host genome in these four tumors is likely to be involved in the risk of developing lymphoma simultaneously with integration sites [15]. Thus, the significance of integration sites in lymphoma development after BLV infection has not yet been fully elucidated.

Therefore, to clarify the relationships between BLV provirus integration, clonal expansion, and disease progression in BLV-infected cattle, it is necessary to characterize the integration sites using blood samples collected from a BLV-infected cow at different stages i.e. from asymptomatic to the terminal stages of leukemia/lymphoma. In this study, we collected blood samples classified into three stages such as (Stage I, polyclonal stage; Stage II, polyclonal toward oligoclonal stage; Stage III, oligoclonal stage) from a naturally-infected Holstein dairy cow at three time points, and successfully monitored the alternation of the BLV integration sites using our BLV proviral DNA-capture sequencing method, which is a target enrichment high-throughput sequencing system for characterization of BLV integration sites [14]. This is the first report to illustrate the time course of clonal expansion after massive depletion of cells carrying BLV proviral clones and sequential changes of integration sites in naturally infected BLV individuals progressing from the premalignant stage to the terminal disease.

## Results

### Chronic stage of samples were collected from a BLV naturally-infected Holstein cow

Blood samples were collected at three time points from a Holstein dairy cow naturally infected by BLV (first: 2017.05.23, second: 2017.11.02, last time: 2018.02.15 Leukemia onset). Table 1 shows the number of White blood cells (WBC) and lymphocytes and BLV proviral load (PVL) in stages I, II, and III, respectively. The lymphocyte count in Stage I was significantly higher than that in Normal cattle, while that in Stage II was subsequently reduced to one-third of the normal value. When Stage III was finally followed to develop lymphoma and death, the lymphocyte count drastically increased. PVL were analyzed by BLV-CoCoMo-qPCR-2 and were 64,554, 4463, and 83,163 copies per 10^5^ cells in Stages I, II, and III, respectively, in the same manner as lymphocyte counts. Thus, the three stages from the same cattle were classified according to the EC-leukosis key [16]: stage I, BLV-infected but clinically normal cattle with lymphocytosis; Stage II, BLV-infected but clinically normal cattle with a decreased lymphocyte count; and Stage III, BLV-infected with lymphoma (Table 1).

**Table 1:** Clinical information used in this study

Sample name	Sample colletion	Age (moon)	White blood cell count (/µL)	Lymphocyte count(/µL)	BLV proviral load (copies/10^5^cells)^a^	BLV antibody^b^	Clinical stage^c^
Stage I	2017.05.23	49	15,100	10,100	64,554	+	Lymphocytic
Stage II	2017.11.02	54	6800	2700	4463	+	< Normal
Stage III	2018.02.15	58	129,800	88,600	83,163	+	Leukemia

^a^The proviral load (expressed as the copy number per 10^5^ cells) was evaluated by BLV-CoCoMo-qPCR-2

^b^ELISA was performed using an anti-BLV ELISA kit, according to the manufacturer’s instructions (JNC Inc., Tokyo, Japan). +: Positive for anti-BLV antibodies

^c^The clinical stage of BLV infection was evaluated according to the lymphocyte count (per 1 µL) and age of the animal [14]

### Results of BLV proviral DNA-capture-seq

We clarified the BLV integration sites from Stages I–III using BLV proviral DNA-capture-seq, which is a target-enrichment high-throughput sequencing system for characterization of BLV integration sites [14]. The DNAs molecules were fragmented to an average length of approximately 600 bp to generate DNA libraries. Because virus-host chimeric reads generally contain the BLV long terminal repeat (LTR) sequence and BLV LTR contains 531 nucleotides, the BLV probes used in this study (Additional file 1) would correctly capture the virus-host chimeric fragments. After enrichment of the BLV genome, the prepared library was analyzed using an Illumina MiSeq sequencing system. Paired-end reads were first aligned against the BLV reference FLK-BLV (EF600696), mapped to the cow host reference genome (Bos_taurus_UMD_3.1/bosTau6), and visualized using Integrative genomics viewer (IGV) (Additional file 2). We obtained 4,399,364, 3,730,443, and 4,343,678 reads from Stage I, II, and III DNA, respectively. Of these reads, 3,544,097 (80.5%), 3,042,346 (81.6%), and 3,499,444 (80.6%) were aligned to the BLV provirus LTR, *gag*, or *tax* regions. These data indicate that target enrichment high-throughput sequencing was successful in all samples.

### Confirmation of the Integration Sites

**Fig. 1:**
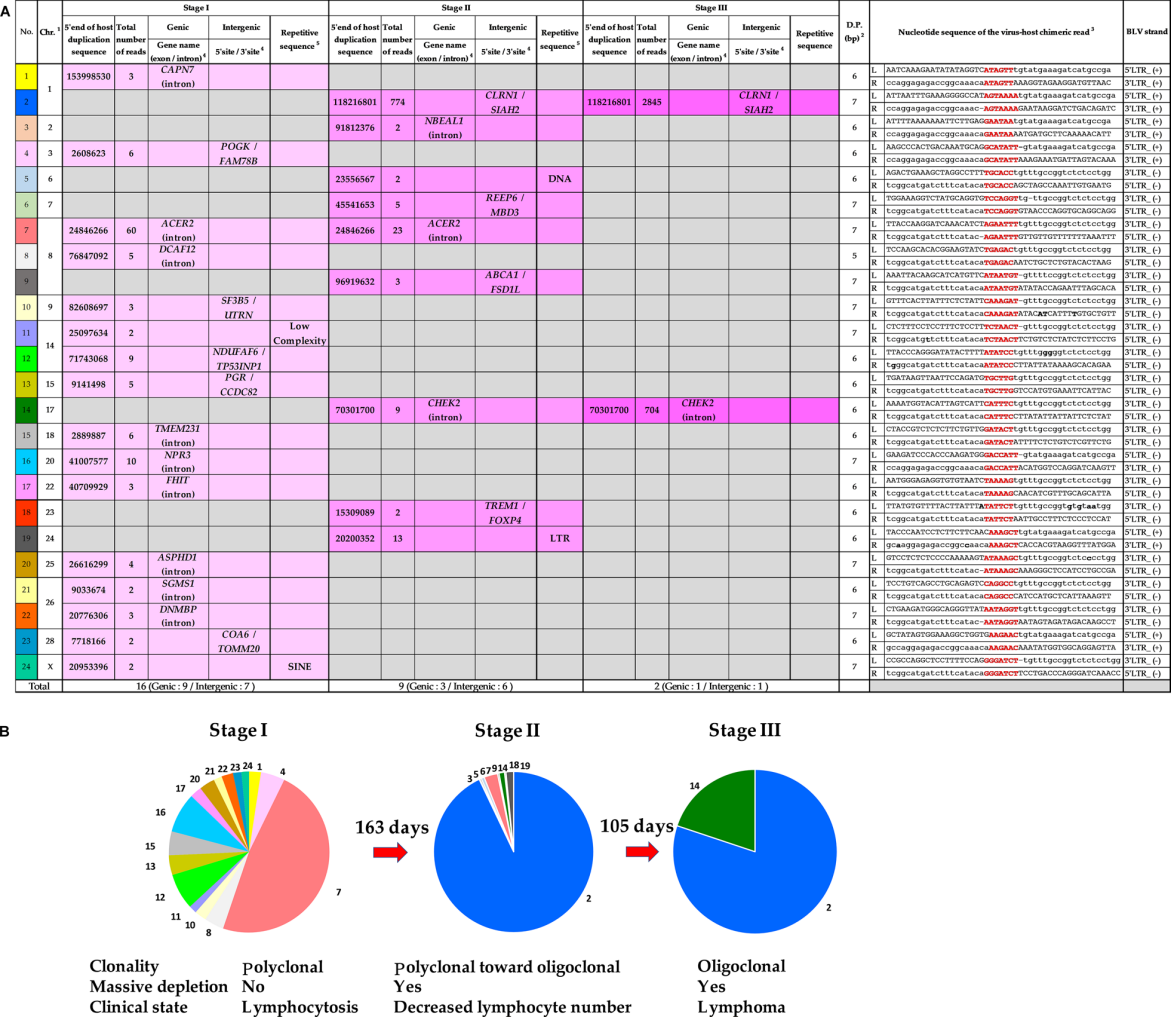
Change of the BLV integration sites during the course of disease progression in a BLV naturally-infected cow. **A** Summary of the integration sites detected in Stage I, Stage II, and Stage III, respectively. ^1^Chr, Chromosome. ^2^D.P, Duplicated Nucleotide. ^3^L, left read; R, right read; red color, duplicated nucleotide sequence; upper case, nucleotide sequence of the cattle; lower case, nucleotide sequence of the BLV; bold letter, different sequence compared to the reference sequence; –, deleted sequence compared to the reference sequence. ^4^*CAPN7*, calpain 7; *ACER2*, alkaline ceramidase 2; *DCAF12*, DDB1 and CUL4 associated factor 12; *TMEM231*, transmembrane protein 231; *NPR3*, natriuretic peptide receptor 3; *FHIT*, fragile histidine triad diadenosine triphosphatase; *ASPHD1*, aspartate beta-hydroxylase domain containing 1; *SGMS1*, sphingomyelin synthase 1; *DNMBP*, dynamin binding protein; *POGK*, pogo transposable element derived with KRAB domain; *FAM78B*, family with sequence similarity 78 member B; *SF3B5*, splicing factor 3b subunit 5; *UTRN*, utrophin; *NDUFAF6*, NADH:ubiquinone oxidoreductase complex assembly factor 6; *TP53INP1*, tumor protein p53 inducible nuclear protein 1; *PGR*, progesterone receptor; *CCDC82*, coiled-coil domain containing 82; *COA6*, cytochrome c oxidase assembly factor 6; *TOMM20*, translocase of outer mitochondrial membrane 20; *NBEAL1*, neurobeachin like 1; *CHEK2*, checkpoint kinase 2; *CLRN1*, clarin 1; *SIAH2*, siah E3 ubiquitin protein ligase 2; *REEP6*, receptor accessory protein 6; *MBD3*, methyl-CpG binding domain protein 3; *ABCA1*, ATP binding cassette subfamily A member 1; *FSD1L*, fibronectin type III and SPRY domain containing 1 like; *TREM1*, triggering receptor expressed on myeloid cells 1; *FOXP4*, forkhead box P4. ^5^Low Complexity, Low complexity repeats; SINE, Short interspersed nuclear elements; DNA, DNA repeat elements; LTR, Long terminal repeat elements. **B** Pie charts showing the relative abundance of each sample at various disease progression from a BLV-infected cow in Stages I, II, and III, respectively. The numbers of integration site represent each of the detected clone in Fig. 1 were described. State of clonal expansion of cells carrying the integration site and massive depletion of cells carrying the integration site, and clinical stage of disease progression of Stages I, II, and III from one BLV-infected cow, respectively, indicated

BLV was integrated at 16, 9, and 2 distinct integration sites into the host genome in Stages I, II, and III, respectively (Fig. 1A). We observed 6 or 7 bp of short duplicated host genome sequences generated during integration around the BLV provirus integration in all 24 integrated sites (red in Fig. 1A), as visualized using IGV. Furthermore, we determined the strand orientation of the BLV genome to the host genome for all 24 integrated sites (Fig. 1A). To confirm the integration sites detected in the IGV profile, we selected three major integration sites in Stage I [Chromosome (Chr) 8], Stage II (Chr 8 and Chr 1), and Stage III (Chr 1 and Chr 17), and subjected them to PCR using primers set for the host genome and BLV genome (Additional file 1).

**Fig. 2:**
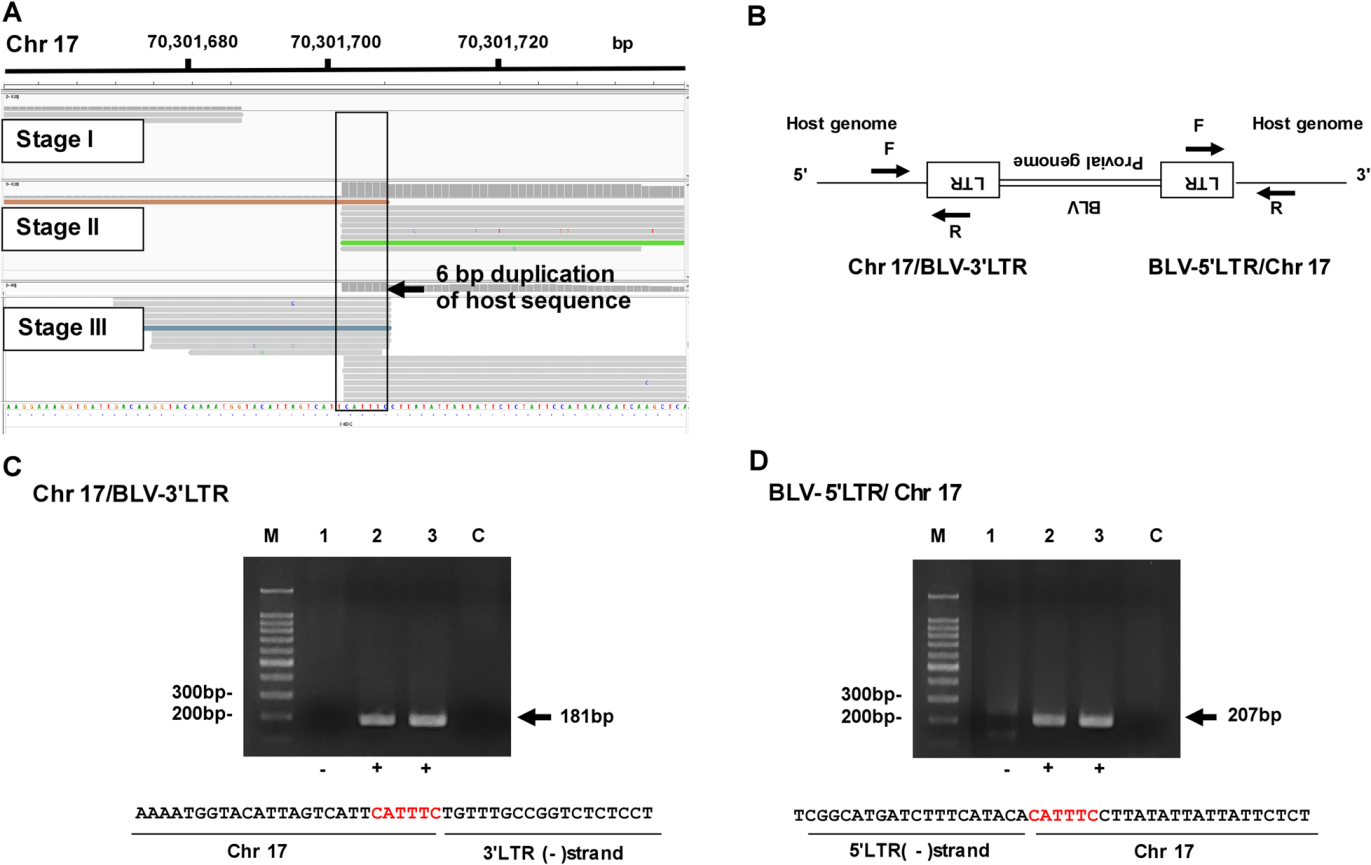
Visualization of the integration site identified on Chr 17. **A** Visualization of NGS reads detected on IGV profile. **B** Schematic diagram to detect both Chr 17/BLV-3′LTR chimeric fragment and BLV-5′LTR/Chr 17 chimeric fragment. Arrows indicate the position of primers. **C** Confirmation of the Chr 17/BLV-3′LTR chimeric fragment using conventional PCR and Sanger sequence. Arrow indicates the PCR product of the Chr 17/BLV-3′LTR chimeric read. M, 100 bp DNA Ladder marker (MIXELL Inc, Tokyo, Japan); 1, Stage I; 2, Stage II; 3, Stage III; C, no-template DNA-negative control (water substituted for DNA template). Six nucleotides duplication of host sequence (CATTTC) was detected by Sanger sequence. **D** Confirmation of the BLV- 5′LTR/Chr 17 chimeric fragment using conventional PCR and Sanger sequence. Arrow indicates the PCR product of the BLV- 5′LTR/Chr 17 chimeric fragment. M, 100 bp DNA Ladder marker (MIXELL Inc, Tokyo, Japan); 1, Stage I; 2, Stage II; 3, Stage III; C, no-template DNA-negative control (water used as the substitute for DNA template). 6 nucleotides duplication of host sequence (CATTTC) colored in red was detected by Sanger sequence

The IGV profile mapped to the bovine Chr 17 genome is shown in Fig. 2A. In the case of Chr 17, the integration site was detected in Stages II and III only. Six nucleotide duplications of the host sequence (CATTTC) were detected in the IGV profile. Figure 2B shows a schematic diagram to detect both the Chr 17/BLV-3′LTR chimeric fragment and the BLV-5′LTR/Chr 17 chimeric fragment. Figure 2C, D show the results of PCR amplification on Chr 17. Following PCR analysis, we obtained PCR products of 181 bp and 207 bp in Stages II and III, but not in Stage I (Fig. 2C, D). After sequencing both the 5′ and 3′ sites, we confirmed six nucleotides of the host sequence (CATTTC) (Fig. 2C, D). In addition, to confirm the integration sites with greater accuracy, we performed inverse PCR using Stage III DNA and obtained the same results as the IGV profile, successfully identified one integration site on Chr 17, and observed six nucleotides of the host sequence (CATTTC) directly flanking the 5′ and 3′ ends of the BLV provirus (data not shown).

**Fig. 3:**
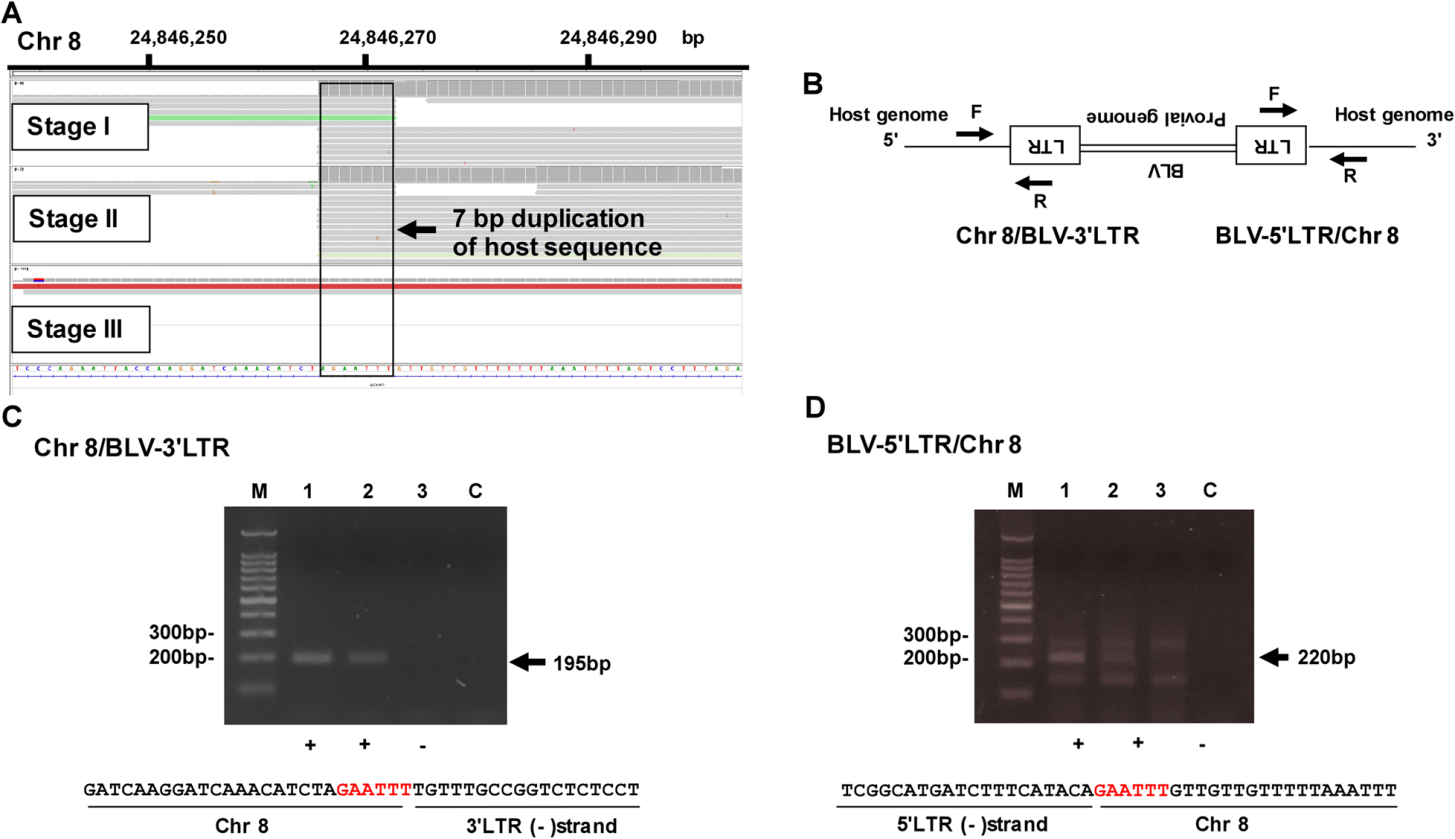
Visualization of the integration site identified on Chr 8. **A** Visualization of NGS reads detected on IGV profile. **B** Schematic diagram to detect both Chr 8/BLV-3′LTR chimeric fragment and BLV-5′LTR/Chr 8 chimeric fragment. Arrows indicate the position of primers. **C** Confirmation of Chr 8/BLV- 3′LTR chimeric fragment using conventional PCR and Sanger sequence. Arrow indicates the PCR product of the Chr 8/BLV-3′LTR chimeric fragment. M, 100 bp DNA Ladder marker (MIXELL Inc, Tokyo, Japan); 1, Stage I; 2, Stage II; 3, Stage III; C, no-template DNA-negative control (water substituted for DNA template). Six nucleotides duplication of host sequence (GAATTT) was detected by Sanger sequence. **D** Confirmation of BLV-5′LTR/Chr 8 chimeric fragment using conventional PCR and Sanger sequence. Arrow indicates the PCR product of the BLV- 5′LTR/Chr 8 chimeric fragment. M, 100 bp DNA Ladder marker (MIXELL Inc, Tokyo, Japan); 1, Stage I; 2, Stage II; 3, Stage III; C, no-template DNA-negative control (water substituted for DNA template). 6 nucleotides duplication of host sequence (GAATTT) colored in red was detected by Sanger sequence

The IGV profile mapped to the bovine Chr 8 genome is shown in Fig. 3A. In the case of Chr 8, the integration site was detected in Stages I and II, but not in Stage III. Seven nucleotides of the host sequence (AGAATTT), directly flanking the 5′ and 3′ ends of the BLV provirus, were detected in the IGV profile. Figure 3B shows a schematic diagram to detect both the Chr 8/BLV-3′LTR chimeric fragment and the BLV- 5′LTR/Chr 8 chimeric fragment. Figure 3C, D show the results of PCR amplification on Chr. 8. Following PCR analysis, we obtained PCR products of 195 bp and 220 bp in Stage I and Stage II, but not in Stage III (Fig. 3C, D). After sequencing both the 5′ and 3′ sites, we confirmed six nucleotides (GAATTT) (Fig. 3C, D).

**Fig. 4:**
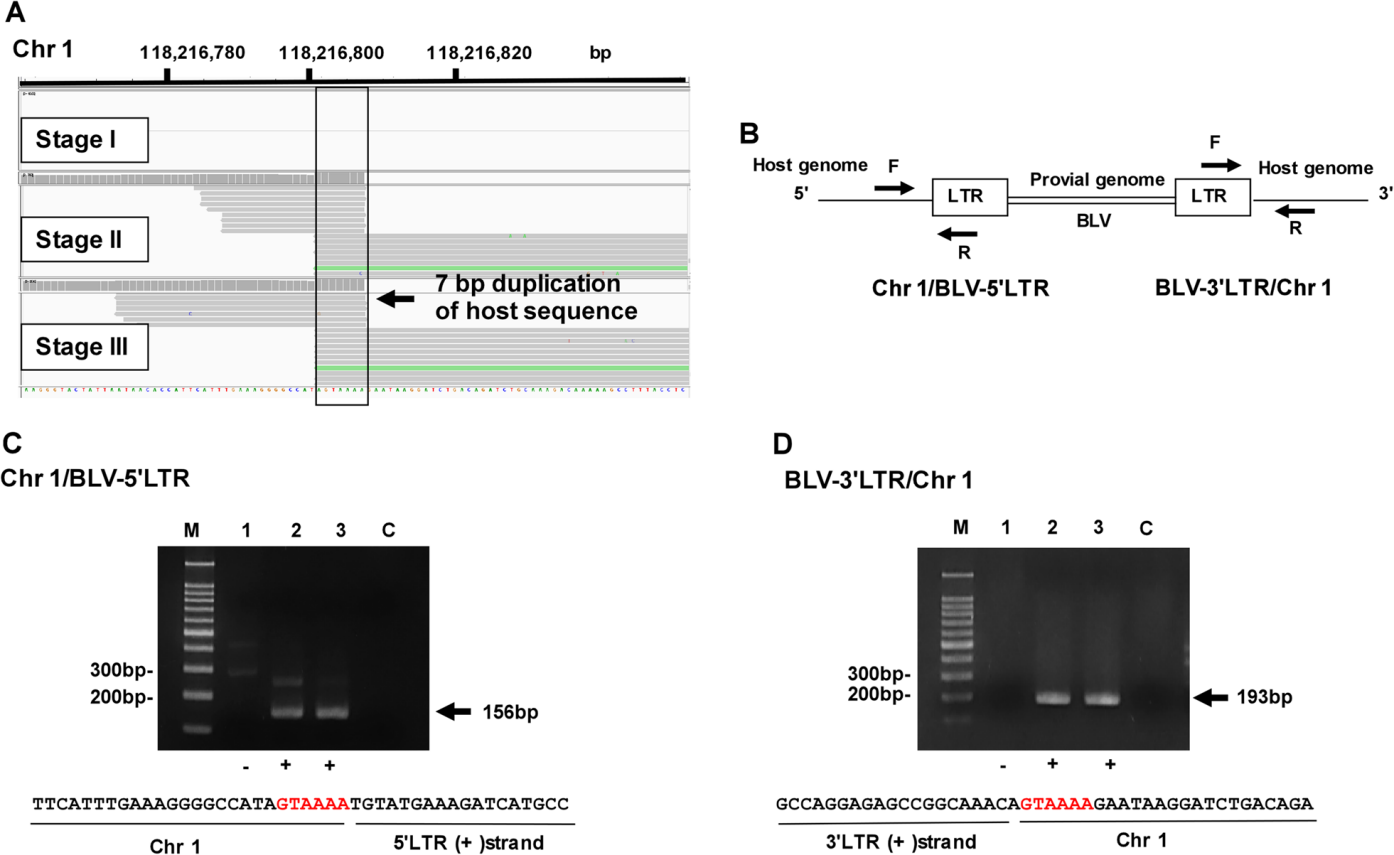
Visualization of the integration site identified on Chr 1. **A** Visualization of NGS reads detected on IGV profile. **B** Schematic diagram to detect both Chr 1/BLV-5′LTR chimeric fragment and BLV-3′LTR/Chr 1 chimeric fragment. Arrows indicate the position of primers. **C** Confirmation of the Chr 1/BLV-5′LTR chimeric fragment using conventional PCR and Sanger sequence. Arrow indicates the PCR product of the Chr 1/BLV-5′LTR chimeric read. M, 100 bp DNA Ladder marker (MIXELL Inc, Tokyo, Japan); 1, Stage I; 2, Stage II; 3, Stage III; C, no-template DNA-negative control (water substituted for DNA template). Six nucleotides duplication of host sequence (GTAAAA) was detected by Sanger sequence. **D** Confirmation of the BLV-3′LTR/Chr 1 chimeric fragment using conventional PCR and Sanger sequence. Arrow indicates the PCR product of the BLV-3′LTR/Chr 1 chimeric fragment. M, 100 bp DNA Ladder marker (MIXELL Inc, Tokyo, Japan); 1, Stage I; 2, Stage II; 3, Stage III; C, no-template DNA-negative control (water substituted for DNA template). 6 nucleotides duplication of host sequence (GTAAAA) colored in red was detected by Sanger sequence

The IGV profile mapped to the bovine Chr 1 genome is shown in Fig. 4A. In the case of Chr 1, the integration site was detected in Stages II and III, but not in Stage I, 7 nucleotides duplication of the host sequence (AGTAAAA) was also detected in the IGV profile. Figure 4B shows a schematic diagram to detect both the Chr 1/BLV-5′LTR chimeric fragment and the BLV-3′LTR/Chr 1 chimeric fragment. Following PCR analysis, we obtained PCR products of 156 bp and 193 bp in Stages II and Stage III, but not in Stage I (Fig. 4C, D). After sequencing both the 5′ and 3′ sites, we confirmed six nucleotides (GTAAAA) (Fig. 4C, D).

### Clonal expansion and massive depletion of cells carrying distinct BLV Integration Sites

Figure 1A shows a summary of BLV integration sites in Stages I, II, and III, and Fig. 1B shows their pie charts.

In Stage I, 16 distinct proviral integration sites were detected. The integration sites in Stage I were the most abundant among the three stages. Therefore, Stage I was characterized as a polyclonal stage (Fig. 1B). These integration sites were mapped to 12 autosomes and X-chromosomes. Multiple integration sites were mapped to three autosomal sites (chr 8, 14, and 26). Interestingly, 9 (56%) out of 16 distinct integration sites were located within introns of reference sequence (Refseq) genes, such as calpain 7 (*CAPN7*), alkaline ceramidase 2 (*ACER2*), DDB1 and CUL4 associated factor 12 (*DCAF12*), transmembrane protein 231 (*TMEM231*), natriuretic peptide receptor 3 (*NPR3*), fragile histidine triad diadenosine triphosphatase (*FHIT*), aspartate beta-hydroxylase domain containing 1 (*ASPHD1*), sphingomyelin synthase 1 (*SGMS1*), and dynamin binding protein (*DNMBP*). The remaining five (31%) and two (13%) sites were located in intergenic regions and within repetitive sequences, such as low-complexity repeats and short interspersed nuclear elements (SINE). In 15 out of 16 integration sites, the total number of reads was 20 or less, and they subsequently disappeared in Stage II in the later 6 months. In contrast, only one clone in which BLV integration occurred within the intron of *ACER2* gene on Chr 8 and the total number of 60 reads was able to survive until Stage II; however, the read depth of this integration decreased in Stage II and finally disappeared in Stage III.

In Stage II, we identified nine distinct proviral integration sites. Among them, eight integration sites that were not identified in Stage I were newly detected, unlike one site that was inserted in the intron of *ACER2* already identified in Stage I. Contrastingly, 15 integration sites were present in Stage I and were not detected in Stage II, indicating a massive depletion of 15 BLV-infected clones that occurred for 163 days during Stages I and II. Collectively, the lymphocyte count and PVL in Stage II were considerably reduced to one-fourth and one-fifteenth of that in Stage I, respectively. Therefore, stage II clonality was characterized as polyclonal towards the oligoclonal stage, as shown in Fig. 1B. Multiple integration sites were mapped to an autosomal chromosome (chr 8). In seven (78%) out of nine integration sites, the total number of reads was 20 or less and disappeared in Stage III. Among these nine distinct integration sites, three (33%) were located within the introns of Refseq genes, such as neurobeachin-like 1 (*NBEAL1*) and checkpoint kinase 2 (*CHEK2*), including *ACER2* gene. The remaining four (44%) and two (22%) sites were located in intergenic regions and within repetitive sequences, such as DNA repeat elements (DNA) and LTR, respectively. The most common integration site was Chr 1; BLV was integrated into the intergenic region downstream of clarin 1 (*CLRN1)* and upstream of siah E3 ubiquitin protein ligase 2 (*SIAH2).* The read depth of this integration was 774, which rapidly increased in Stage III. Another integration site within the intron of *CKEK2* on Chr 17 was detected in Stage II.

As shown in Table 1, at Stage III, the test subject was diagnosed with lymphoma. Seven integration sites that were present in Stage II, could not be detected in Stage III, indicating that seven BLV-infected clones were eliminated for approximately 3 months during Stage II and Stage III. In contrast, we identified only two distinct proviral integration sites that were generated first at Stage II, indicating that the surviving clones carrying the integration site on Chr 1 and in the intron of *CHEK2* evaded massive clone selection from Stage II to Stage III and provoked polyclonal expansion. The read depth of the two integrations rapidly increased as the total number of reads reached 2845 in Stage III. Another integration site within the intron of *CHEK2* on Chr 17 was detected in stage II. Although the total number of reads of the integration site within the intron of *CHEK2* on Chr 17 was only nine in Stage II, it increased by 78-fold in Stage III.

## Discussion

In this study, we monitored the sequential alternation of BLV integration sites in a BLV naturally-infected Holstein cow from Stages I to III, using proviral DNA-capture-seq previously developed by us [14]. In this study, we identified 16 distinct proviral integration sites in Stage I. As the total reads of these integration sites were low in abundance, cells carrying 15 sites disappeared after 163 days during Stages I and II, and cells carrying one site disappeared after 105 days during Stages II and III. It is conceived as a massive depletion of 16 BLV-infected clones that may occur at Stages II and III. Instead of these 15 proviral integration sites, 8 new integration sites arose alternatively at a low abundance during Stage II. According to Stage III, seven of these integration sites rapidly decreased, indicating that a massive depletion of seven BLV-infected clones may have occurred for 105 days during Stages II and III. These results indicate that most of the infected clones are created during infection and are subsequently depleted before onset because of the host immune response [10]. Our results suggest that BLV may continuously attack host cells from infection to terminal leukemia/lymphoma. Our assumption is supported by a previous report that host negative selection eliminated almost all BLV proviral clones detected at seroconversion in an experimentally infected sheep [10]. In contrast, we showed that cells carrying only two integration sites finally increased in lymphoma onset, indicating that surviving clones escaped from massive depletion of almost all clones and shifted toward clonal expansion. These findings show the same tendency as the previous result [10] that initial replication reveals a polyclonal pattern of viral expansion, and BLV replication shifts to oligoclonal expansion soon after primary infection [10]. The same result was observed for HTLV-1 [17]. This is the first study to report the visualization of the time course of clonal expansion after massive depletion of cells carrying BLV proviral clones in a BLV naturally-infected Holstein cow.

In contrast to previous studies reporting that in HTLV-1 and BLV, proviral integrations in the host genome preferentially occur in transcriptionally active genomic regions near transcriptional start sites and transcription factor-binding sites [10, 17–20], 11 (46%) out of the 24 distinct proviral integration sites identified in this study were found to be located within introns of Refseq genes. Additionally, the remaining nine (37%) and four (17%) sites were located in intergenic regions and within repetitive sequences, respectively. Thus, BLV provirus is more likely to integrate into Refseq gene introns than transcriptionally active genomic regions. This is congruent with the results of a previous study that analyzed four tumors from four cattle with EBL [15]. However, although 10 out of these 11 integration sites were located within introns of Refseq genes and disappeared until Stage III, only cells carrying integration sites within the intron of *CHEK2* on Chr 17 drastically increased in Stage III. In addition, all 24 integration sites were different in this study and from those reported in previous studies where BLV integration sites in EBL cattle were found in Refseq genes including *RPTOR* [14], Family with sequence similarity 92 member A (*FAM92A*), *FAM135B*, Ankyrin 3 (*ANK3*), and uncharacterized genes [15], and in retroelements, such as SINE, LINE, and LTR of the endogenous retrovirus [15, 21]. Our data were obtained from a single, naturally infected BLV cow that progressed from the premalignant stage of lymphoma to the terminal disease. Therefore, further comprehensive studies are necessary to clarify whether provirus-dependent host-gene perturbation at each integration site contributes to the selection of multiple clones or accelerated proliferation of infected cells [13].

Both the 5′ and 3′ sites of the BLV genome comprise an LTR region, which is connected to the *gag* and *tax* genes. Therefore, we used a target enrichment high throughput sequencing system to detect BLV integration sites, as described elsewhere [14], using five biotinylated DNA probes, three for the LTR region, and one for the *gag* and *tax* region, respectively. Contrary to complete proviruses, previous studies identified HTLV-1 and BLV provirus-subtypes. Type-1 defective proviruses contain both 5′ and 3′ LTRs, but lack a section of the proviral sequence that exists between these regions. Conversely, type-2 defective proviruses lack the 5′ LTR, but are never 3′LTR-deficient [13, 22, 23]. Therefore, among our five probes, the LTR-targeting probes captured both complete and type-1 defective proviruses, while the *gag*-specific probes and *tax*-specific probes captured type-2 defective proviruses keeping either the *gag* region or *tax* region. We successfully detected a single BLV proviral integration site on Chr 19 of BLSC-KU1. This site comprised a ± 1.7 kbp deletion that expanded from the C-terminal of the *pol* gene and included the majority of the *env*-gp51 gene [14]. Thus, our five biotinylated DNA probes correctly captured complete and defective proviruses. However, we here chased not to detect defective proviruses; high-throughput DNA sequencing techniques identified 86% of BLV integration sites via both 5′ and 3′ LTR-flanking host sequences (including type 1 defects) and 14% via only 3′ LTR-flanking sequences (type 2 defects) [13]. HTLV-1 integration sites exhibited the same tendency [13, 24]. Therefore, we introduced the following two requirements to identify the integration sites of proviruses: (1) the distinctive 6–7 bp duplication of the host sequence, generated during the integration, must be present and the repeats must overlap when paired-end reads are aligned and mapped to the host reference genome; and (2) the bovine portion of at least one of the “left reads” and “right reads” must align to the same chromosome with a convergent orientation [14]. After imposing these criteria, type 2 defective proviruses could not be identified. An integrated BLV provirus, defective at the 5′ end, was present in cattle with persistent lymphocytosis [13, 15]. These defects have also been reported in HTLV-1 [24] and HIV-1 [25]. Therefore, further research is required to elucidate the integration site of the type-2 defective proviruses and the proliferation of cells that contain these proviruses, to ultimately explicate the mechanism of BLV provirus integration.

When retroviruses integrate into the host cellular genome, short repetitive sequences are generated adjacent to both LTRs by viral integrase [26]. In the case of HIV-1, integration introduces a 5-bp repeat sequence [25, 26], HTLV-1 Virus a 6-bp repeat sequence [24, 27]. In this study, we found three major integration sites: Stage I (Chr 8), Stage II (Chr 1 and Chr 8), and Stage III (Chr 1 and Chr 17). To confirm these integration sites, we performed inverse PCR using primers set for the genomes of host and BLV. We obtained a 6-bp repeat sequence for all three major integration sites. However, in the IGV profile, we obtained a 7-bp repeat sequence in two major integration sites (Chr 1 and Chr 8). By characterizing the nucleotide sequences, short repetitive sequences in the host cellular genome were mapped to a 7-bp repeat sequence on the IGV profile. According to these results, we could select virus-host chimeric fragments fulfilling the following conditions to determine the integration sites of the proviruses: a distinctive 6–7 bp duplication of the host sequence generated during the integration process should be present. Indeed, 6–7 bp duplication sequences in the host cellular genome were detected in all 24 integrated sites found in this study on the IGV profiles. In addition, we previously identified a 6-bp repeat sequence in two distinct integration sites in Chr 19 and Chr 9 in the bovine B-cell lymphoma lines BLSC-KU1 and BLSC-KU17, respectively [14].

In the chronic stages of infection, HTLV-1 propagates primarily through clonal expansion of infected T cells, resulting in multiple clones of varying abundance, each uniquely identified by their proviral integration sites in the host genome. Following a protracted incubation period, one of these clones expands, leading to the accumulation of malignant cells in the peripheral blood (leukemia) and/or diverse tissues (lymphoma) [17, 28, 29]. Here, our result obtained from a BLV naturally-infected Holstein cow is the first report to demonstrate clonal expansion after massive depletion of cells carrying BLV integration sites by visualizing cells carrying BLV proviral integration sites during a BLV naturally-infected Holstein cow progress from the premalignant stage to the terminal disease. From Stage II to Stage III, we found two major integration sites on Chr 1 and Chr 17, allowing escape from the host immune surveillance. Because these sites were not detected in Stage I, we considered them to be de novo integrations. One of the important outcomes was the detection and confirmation of the BLV proviral integration site within the intron of *CHEK2* gene on Chr 17. The BLV provirus integrates within introns of the *CHEK2* gene, which is considered a candidate cancer driver gene because the *CHEK2* gene is known to be a tumor suppressor gene encoding serine/threonine kinase CHEK2 and is involved in DNA repair in response to DNA damage, cell cycle arrest, and apoptosis [30]. Interestingly, mutations, copy number variation, gene expression, and mutation in the *CHEK2* gene have been linked to a variety of cancers, as described in the COSMIC database (https://cancer.sanger.ac.uk/cosmic). Although the total number of reads of the integration site within the intron of *CHEK2* was only nine in Stage II, it increased by 78-fold in Stage III. This indicates that BLV provirus integration within the introns of the cancer driver gene is affected by either provirus-dependent transcription termination or host gene perturbation [13]. Another finding of our study is that BLV was integrated into the intergenic region downstream of *CLRN1* and upstream of *SIAH2* on Chr 1. Interestingly, provirus integration by the Moloney murine leukemia virus, which induces T-cell lymphomas in a single locus, activates the expression of multiple genes, some of which may be located far from the site of integration [31]. In addition, it appeared that BLV and HTLV-1 integrations deregulated the host cellular 3D chromatin organization through the formation of viral/host chromatin loops *in cis* [32–34]. Stage III lymphoma might be associated with the polyclonal expansion of two integrated, main BLV proviruses. Therefore, further studies are needed to clarify the impact of BLV proviral integration into *CHEK2* gene and the intergenic region between *CLRN1* and *SIAH2*, the host cellular genes reported in this study.

## Materials and methods

### Sample collection, extraction of genomic DNA and diagnosis

Blood samples were obtained three times (Stages I, II, and III) from a naturally infected Holstein dairy cow in a breeding farm located in Chiba Prefecture, Japan (Table 1). Serum samples were also obtained from the same cow.

BLV infection was evaluated using BLV-CoCoMo-qPCR-2 (RIKEN Genesis, Kanagawa, Japan) with THUNDERBIRD Probe qPCR Mix (TOYOBO Co., Ltd., Osaka, Japan) [35–37], and an anti-BLV antibody enzyme-linked immunosorbent assay (ELISA) kit (JNC Inc., Tokyo, Japan). The subclinical stage of BLV infection was diagnosed according to the lymphocyte count (cells/µL) and age of each cow (≤ 7500 = normal and ≥ 9500 = lymphocytosis for cows aged 3–4 years; ≤ 6500 = normal and ≥ 8500 = lymphocytosis for cows aged ≥ 4–5 years) [16]. BLV-infected but clinically and hematologically normal cows were defined as asymptomatic cattle, whereas BLV-infected but clinically normal cattle showing an increase in the number of apparently normal B lymphocytes were defined as PL cows. Subsequently, lymphoma in cattle was diagnosed based on gross and histological observations. WBC and lymphocytes was measured using an automated veterinary hematology analyzer (MEK-6550 Celltac α, Nihon Kohden, Tokyo, Japan).

This study was approved by the animal ethics committee and the animal care and use of the RIKEN Animal Experiments Committee (Approval Number H29-2-104).

Genomic DNA was extracted from ethylenediaminetetraacetic acid (EDTA)-treated blood samples according to the protocol provided with Wizard Genomic DNA Purification Kit (Promega Corporation, Tokyo, Japan).

### BLV proviral DNA-capture sequencing (BLV proviral DNA-capture-seq) method

To detect the BLV integration sites, we used BLV proviral DNA capture-seq previously developed by us [14]. The cleaned sequencing reads were mapped to the FLK-BLV subclone pBLV913 (GenBank accession number EF600696) with or without the bovine reference genome (Bos_taurus_UMD_3.1/bosTau6) using the BWA-MEM algorithm. High-throughput sequencing was used to map and quantify the insertion sites of the provirus to monitor clonality of the BLV-infected cell population. The aligned reads were visualized using IGV.

### PCR amplification and sequencing of virus-host chimeric fragments

To confirm the presence of virus-host chimeric fragments, nucleotide sequences were amplified and sequenced using primers set to the host genome and BLV genome (Additional file 1), as previously described elsewhere [14].

### Inverse PCR and sequencing

Inverse PCR was performed as described previously with some modifications [21, 38, 39]. Purified genomic DNA (5 µg) was digested with a restriction enzyme (SacI or BamHI) and purified. Digested DNA was self-ligated using a DNA Ligation Kit (Ligation high Ver.2) (TOYOBO Co., Ltd.). The circulated DNA was used as a template for inverse PCR using the primer pair LTR end R2/LTR end F3 or Tax end R2/Tax end F2 (Additional file 1). PCR was conducted using PrimeSTAR GXL DNA Polymerase (Takara Bio Inc., Shiga, Japan) following the manufacturer’s instructions. PCR products were electrophoresed on agarose gels and purified using a FastGene Gel/PCR Extraction Kit (NIPPON Genetics Co., Ltd. Tokyo, Japan). Sequences were determined with ABI PRISM BigDye Terminator v3.1 Cycle Sequencing Kits (Applied Biosystems, Foster City, CA, USA) with the primer pair LTR end R3/LTR end F4 or Tax end R3/Tax end F3 (Additional file 1).

## Supplementary Information

**Additional file 1.** Probes for NGS and PCR primers.

**Additional file 2: Figure S1.** Visualization of paired-end short read sequences of Stages I, II, and III mapped to the BLV reference FLK-BLV sequence (NCBI accession number EF600696). Horizontal lines in the schematic structure of the biotinylated probe targeting region at the top indicate biotinylated probes used in this study.

## Data Availability

All available data are presented in this manuscript.
