# Supplementary material for: Visualization of clonal expansion after massive depletion of cells carrying the bovine leukemia virus (BLV) integration sites during the course of disease progression in a BLV naturally-infected cow: a case report
Source: Retrovirology. 2022 Nov 3;19:24. doi: 10.1186/s12977-022-00609-0 (PMC9635170; doi:10.1186/s12977-022-00609-0)
Supplement: Supplementary file 1 — Additional file 1. Probes for NGS and PCR primers. [file 12977_2022_609_MOESM1_ESM.pdf]

Additional file 1: Probes for NGS and PCR primer.

Methods	Probe / Primer name	Primer Sequence, 5' to 3'	Probe / primer position	GenBank Reference Sequence
NGS	Probe_LTR_1	TGTATGAAAGATCATGCCGA-CCTAGGCGCCGCCACCGCCC-CCTAGGCGCCGCCACCGCCC-CAGCTGCCAGAAAAGCTGGT-GACGGCAGCTGGTGGCTAGA	1-100(5'LTR), 8190-8289(3'LTR)	EF600696.1
	Probe_LTR_2	CACCTGCTGATAAATTAATA-AAATGCCGGCCCTGTCGAGT-TAGCGGCACCAGAAGCGTTC-TTCTCCTGAGACCCTCGTGC-TCAGCTCTCGGTCCTGAGCT	161-260(5'LTR), 8350-8449(3'LTR)	EF600696.1
	Probe_LTR_3	CGAGACCTTCTGGTCGGCTA-TCCGGCAGCGGTCAGGTAAG-GCAAACCACGGTTTGGAGGG-TGGTTCTCGGCTGAGACCAC-CGCGAGCTCTATCTCCGGTC	271-370(5'LTR), 8460-8559(3'LTR)	EF600696.1
	Probe_gag_1	CCACCCTAAACGAAGTGCTC-TCAAACGATGGGGGCGCCCC-GGGTGCATCGGCCCCAGAAG-AACAACCCCCCCCTTATGAC-CCCCCCGCCGTTTTGCCAAT	861-960	EF600696.1
	Probe_tax_1	CCCCTTATCCAAACGCCCGG-CCTGTCTTGGTCTGTCCCCG-CGATCGACCTATTCCTAACC-GGTCCCCCTTCCCCATGCGA-CCGGTTACACGTATGGTCCA	7941-8040	EF600696.1
Inverse PCR	LTRendR2	CTGGTGCCGCTAACTCGACAG	212-192	EF600696.1
	LTRendF3	GCGTTCTTCTCCTGAGACCCTC	215-236	EF600696.1
	TaxendR2	AGAGTCCACGTGGAGACGGTC	8584-8564	EF600696.1
	TaxendF2	AAGGGCGTCTGGCTTGCACCCG	8612-8633	EF600696.1
Sequencing of the inverse PCR products	LTRendR3	TACGGGGATTCTAGCCACCAG	109-89	EF600696.1
	LTRendF4	GACCCTCGTGCTCAGCTCTCG	230-250	EF600696.1
	TaxendR3	GTGGAGACGGTCAGAGGACCG	8575-8555	EF600696.1
	TaxendF3	GTCTGGCTTGCACCCGCGTTTG	8618-8639	EF600696.1
PCR amplification and sequencing of viral-host junctions (Chr1_BLV5'LTR)	Chr1_F2	AGGGTACTATTAATAACACCATTC	Chr1 : 117278334-117278357	NC_037328.1
	LTRendR3	TACGGGGATTCTAGCCACCAG	109-89	EF600696.1
PCR amplification and sequencing of viral-host junctions (Chr1_BLV3'LTR)	Chr1_R2	TCCCTAGACATAGTCATGCCTC	Chr1 : 117278464-117278443	NC_037328.1
	TaxendF3	GTCTGGCTTGCACCCGCGTTTG	8618-8639	EF600696.1
PCR amplification and sequencing of viral-host junctions (Chr8_BLV5'LTR)	Chr8_R1	GTCACTGGTGTCGTGCCAGTGC	Chr8 : 24881129-24881108	NC_037335.1
	LTRendR3	TACGGGGATTCTAGCCACCAG	109-89	EF600696.1
PCR amplification and sequencing of viral-host junctions (Chr8_BLV3'LTR)	Chr8_F1	TCACAGTTAAGAATTCCAATCTG	Chr8 : 24880933-24880956	NC_037335.1
	TaxendF3	GTCTGGCTTGCACCCGCGTTTG	8618-8639	EF600696.1
PCR amplification and sequencing of viral-host junctions (Chr17_BLV5'LTR)	Chr17_R	GTTGCAATGTCAAACACAACTG	Chr17 : 68009192-68009171	NC_037344.1
	LTRendR3	TACGGGGATTCTAGCCACCAG	109-89	EF600696.1
PCR amplification and sequencing of viral-host junctions (Chr17_BLV3'LTR)	Chr17_F	TCAAGTCACCAAGTTGCACATG	Chr17 : 68009023-68009044	NC_037344.1
	TaxendF3	GTCTGGCTTGCACCCGCGTTTG	8618-8639	EF600696.1
