# Supplementary figures and images for: Visualization of clonal expansion after massive depletion of cells carrying the bovine leukemia virus (BLV) integration sites during the course of disease progression in a BLV naturally-infected cow: a case report
Source: Retrovirology. 2022 Nov 3;19:24. doi: 10.1186/s12977-022-00609-0 (PMC9635170; doi:10.1186/s12977-022-00609-0)

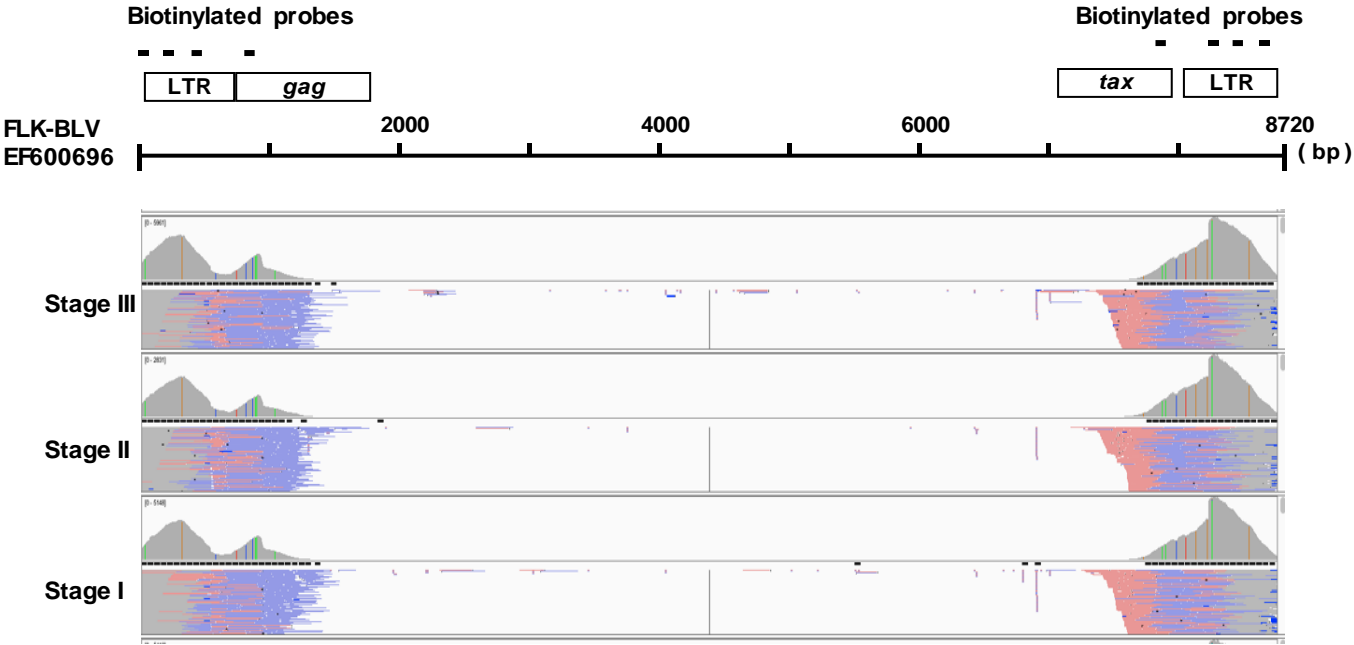

Supplement: Supplementary file 2 — Additional file 2: Figure S1. Visualization of paired-end short read sequences of Stages I, II, and III mapped to the BLV reference FLK-BLV sequence (NCBI accession number EF600696). Horizontal lines in the schematic structure of the biotinylated probe targeting region at the top indicate biotinylated probes used in this study. [file 12977_2022_609_MOESM2_ESM.pdf]
